# Egr2-independent, Klf1-mediated induction of PD-L1 in CD4^+^ T cells

**DOI:** 10.1038/s41598-018-25302-1

**Published:** 2018-05-04

**Authors:** Shuzo Teruya, Tomohisa Okamura, Toshihiko Komai, Mariko Inoue, Yukiko Iwasaki, Shuji Sumitomo, Hirofumi Shoda, Kazuhiko Yamamoto, Keishi Fujio

**Affiliations:** 1 Department of Allergy and Rheumatology, Graduate School of Medicine, The University of Tokyo, 7-3-1 Hongo, Bunkyo-ku, Tokyo, 113-8655 Japan; 2 Max Planck–University of Tokyo Center for Integrative Inflammology, The University of Tokyo, 4-6-1 Komaba, Meguro-ku, Tokyo, 153-8505 Japan; 3 Laboratory for Autoimmune Diseases, Center for Integrative Medical Sciences, RIKEN, 1-7-22 Suehiro-cho, Tsurumi-ku, Yokohama, Kanagawa 230-0045 Japan; 4 Department of Functional Genomics and Immunological Diseases, Graduate School of Medicine, The University of Tokyo, 7-3-1 Hongo, Bunkyo-ku, Tokyo 113-8655 Japan

## Abstract

Programmed death ligand 1 (PD-L1)-mediated induction of immune tolerance has been vigorously investigated in autoimmunity and anti-tumor immunity. However, details of the mechanism by which PD-L1 is induced in CD4^+^ T cells are unknown. Here, we revealed the potential function of Klf1 and Egr2-mediated induction of PD-L1 in CD4^+^ T cells. We focused on the molecules specifically expressed in CD4^+^CD25^−^LAG3^+^ regulatory T cells (LAG3^+^ Tregs) highly express of PD-L1 and transcription factor Egr2. Although ectopic expression of Egr2 induced PD-L1, a deficiency of Egr2 did not affect its expression, indicating the involvement of another PD-L1 induction mechanism. Comprehensive gene expression analysis of LAG3^+^ Tregs and *in silico* binding predictions revealed that Krüppel-like factor 1 (Klf1) is a candidate inducer of the PD-L1 gene (*Cd274*). Klf1 is a transcription factor that promotes β-globin synthesis in erythroid progenitors, and its role in immunological homeostasis is unknown. Ectopic expression of Klf1 induced PD-L1 in CD4^+^ T cells through activation of the PI3K-mTOR signaling pathway, independent of STATs signaling and Egr2 expression. Our findings indicate that Klf1 and Egr2 are modulators of PD-L1-mediated immune suppression in CD4^+^ T cells and might provide new insights into therapeutic targets for autoimmune diseases and malignancies.

## Introduction

The pathological bases of autoimmune diseases such as rheumatoid arthritis (RA) and systemic lupus erythematosus (SLE) have gradually been elucidated^1,2^. Vigorous analyses of cytokines and inhibitory cell surface molecules of immune cells have led to novel treatment methods. Tumor necrosis factor-α (TNF-α) inhibitors, antibodies against the interleukin-6 (IL-6) receptor and cytotoxic T lymphocyte associated protein-4 (CTLA-4) immunoglobulin (Ig) fusion protein have been widely used in the clinic^3^. In inflammatory conditions, T cell responses are regulated both positively and negatively by cell surface molecules. CD28/CD80 (B7-1), CD28/CD86 (B7-2), inducible costimulator (ICOS) and its ligand ICOSL stimulate immune responses. In contrast, immune responses are inhibited by binding of CTLA4/CD80, CTLA4/CD86 and lymphocyte activation gene 3 (LAG3)/MHC class II^4^. Recently, a B7 family co-inhibitory molecule, Programmed death-ligand 1 (PD-L1, encoded by *Cd274*), belonging to the CD28 receptor family^5^, has become a subject of active investigation. Engagement of PD-1 by PD-L1 or PD-L2 transduces a signal that inhibits T-cell proliferation, cytokine production, and cytolytic function. PD-L1 and PD-L2 have marked structural similarities, but they display different expression patterns. Although the expression of PD-L2 is restricted to activated dendritic cells and macrophages^6^, PD-L1 is expressed by activated CD4^+^ T cells, CD8^+^ T cells, natural killer cells, activated monocytes, myelocytes and CD4^-^CD8^-^ (double negative: DN) T cells in the thymus. PD-L1 is also expressed in non-hematopoietic organs such as heart, lung, spleen, thymus and kidney^7^. High expression of PD-L1 has been observed in various tumors including lung cancer and pancreatic cancer^7^.

Whereas PD-L1 is induced by stimulation of the T cell receptor (TCR)^8^, CD4^+^ regulatory T cells (Tregs) constitutively express PD-L1 in the steady state^9,10^. Tregs play a major role in maintaining immune tolerance and are divided into two types: those emerging from the thymus and those induced in the periphery. CD4^+^CD25^+^ Tregs (CD25^+^ Tregs) mainly emerge from the thymus and express the transcription factor forkhead protein 3 (Foxp3) as a master regulator gene (*Foxp3*)^11^. CD25^+^ Tregs inhibit effector cell proliferation and cytokine production^8,12^ and directly suppress B cell activation via PD-L1^13^. Immunological homeostasis is thought to be maintained by CD25^+^ Tregs in concert with peripherally induced Foxp3-independent Tregs. We reported CD4^+^CD25^−^ Tregs that characteristically express LAG3 and Early growth response gene 2 (Egr2) and produce immune suppressive cytokine IL-10^14,15^. Egr2 is a zinc-finger transcription factor that plays an important role in the maintenance of T cell anergy by negatively regulating T cell activation^16^. We previously reported that forced expression of Egr2 in naïve T cells induces LAG3, IL-10, and transcription factor Blimp-1, indicating that Egr2 confers the phenotype of CD4^+^CD25^−^LAG3^+^ Tregs (LAG3^+^ Tregs) on CD4^+^ T cells^14,17^. LAG3^+^ Tregs develop in the periphery and exert their suppressive activities in a Foxp3-independent manner^14^. LAG3^+^ Tregs also produce large amounts of the inhibitory cytokine transforming growth factor-β3 (TGF-β3), which improves the pathology of lupus in a murine model (MRL-Fas^*lpr/lpr*^)^10^. LAG3^+^ Tregs lacking Egr2 attenuate TGF-β3 production and lose suppressive ability for B cells^10^. Interestingly, LAG3^+^ Tregs express high levels of PD-L1. Moreover, TGF-β3-mediated suppression of B cells is dependent on PD-1 expression on B cells^10^. Those observations indicate that PD-L1 expression on LAG3^+^ Tregs play a critical role in the maintenance of immune tolerance. Taken together, these findings suggest that elucidating the molecular control of the PD-1/PD-L1 axis in CD4^+^ T cells is important for clinical applications targeting costimulatory molecules.

In the current study, we show new functions of transcription factor Krüppel like factor 1 (Klf1) and Egr2 in the induction of PD-L1 in CD4^+^ T cells. Klf1 promotes β-globin synthesis in erythroid progenitor cells and its role in immunological homeostasis has not been investigated. Mechanisms that induce PD-L1 expression have been studied mainly in immune cells and tumor cells, and the induction mechanisms of PD-L1 appear to be dependent on cell type. However, the detailed mechanism by which PD-L1 is induced in CD4^+^ T cells remains to be determined. Moreover, it is important to elucidate the cell type-specific mechanism of PD-L1 induction in CD4^+^ T cells. Here, we reveal that Klf1 and Egr2, both of which are characteristically expressed in PD-L1-expressing LAG3^+^ Tregs, induce PD-L1 in CD4^+^ T cells independently of each other.

## Results

### Egr2 induces PD-L1 expression in CD4^+^ T cells

**Figure 1:**
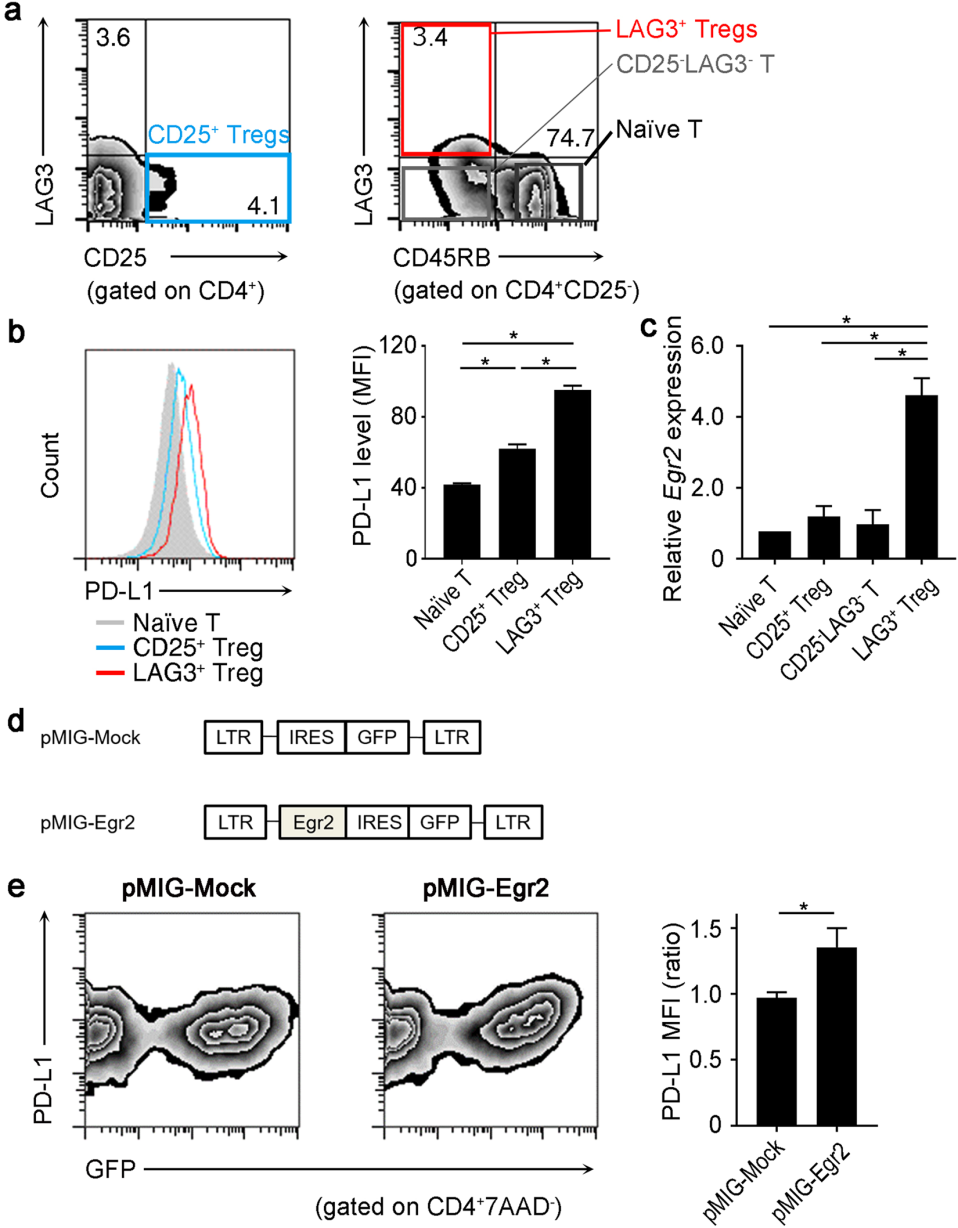
Egr2-mediated PD-L1 induction in CD4^+^ T cells. **(a)** LAG3 expression in splenocytes from C57BL/6 (B6) mice was analyzed by flow cytometry (FCM). CD25^+^ Tregs: CD4^+^CD25^+^ T cells; naïve T: CD4^+^CD25^−^CD45RB^high^ T cells; LAG3^+^ Tregs: CD4^+^CD25^−^CD45RB^low^LAG3^+^ T cells. **(b)** PD-L1 expression in T cell subsets. Histograms are gated on CD4^+^ T cells (left). Data are representative of three independent experiments. Mean fluorescence intensity (MFI) of the indicated T cell subsets is shown (right). (n = 3 per group). *p < 0.001 (Bonferroni’s multiple comparison test) **(c)** Quantitative PCR assessing *Egr2* mRNA expression in the indicated T cell subsets (n = 3 per group). **(d)** Retroviral constructs of pMIG vector for the transduction of Egr2. **(e)** Evaluation of PD-L1 expression in *Egr2* gene-transduced CD4^+^ T cells. MFI ratio represents the PD-L1 MFI signals of GFP negative versus GFP positive. Plots and histogram are the representative of 3 independent experiments.

Constitutive high level expression of PD-L1 in the steady state occurs only in Tregs, such as CD25^+^ Tregs and LAG3^+^ Tregs^9,10^. We first confirmed our previous findings^10,14^ that among CD4^+^ T cell subsets, both Egr2 and PD-L1 were most highly expressed in LAG3^+^ Tregs (Fig. 1a–c). Then, to elucidate whether overexpression of Egr2 induced PD-L1 protein, we utilized the pMIG retroviral vector containing IRES-regulated green fluorescent protein (GFP) as a reporter to generate a pMIG-Egr2 vector (Fig. 1d). As expected, forced expression of Egr2 in naïve CD4^+^ T cells induced PD-L1 protein expression (Fig. 1e). These findings suggest the important role of Egr2 in the induction of PD-L1 in CD4^+^ T cells.

### Egr2 is not essential for the induction of PD-L1 expression in CD4^+^ T cells

**Figure 2:**
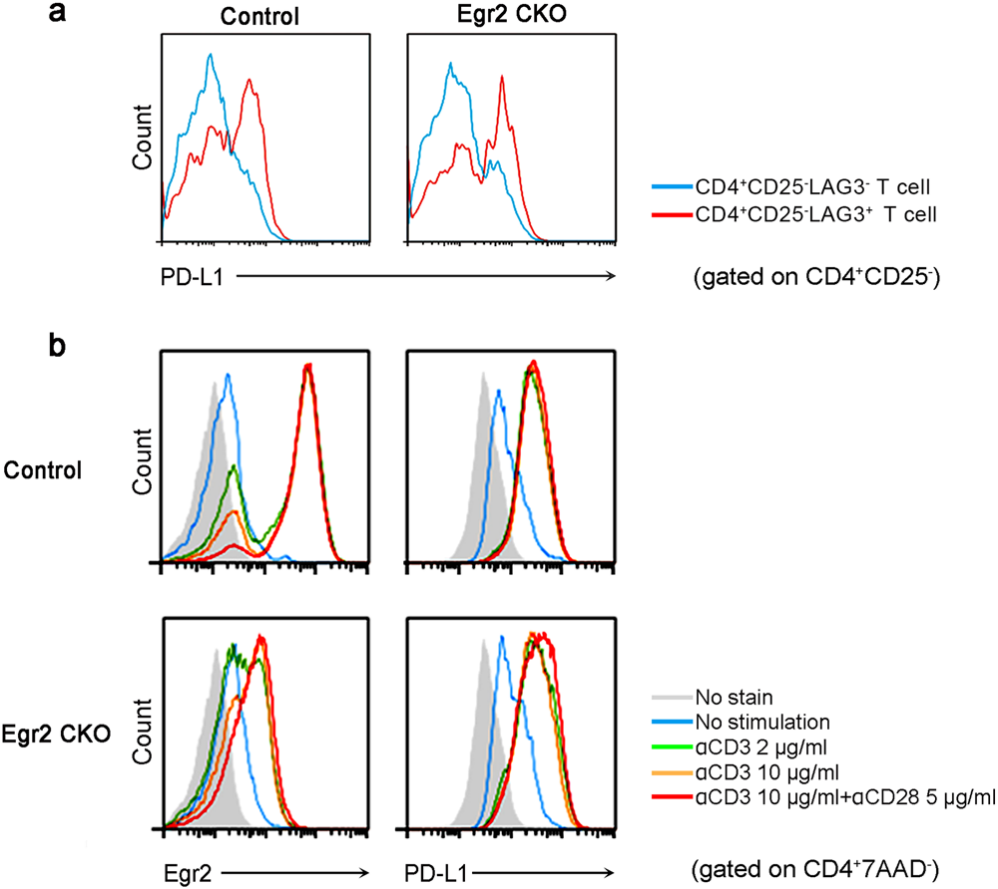
PD-L1 expression in CD4^+^ T cells that were Egr2-deficient. **(a)** PD-L1 expression in splenocytes from T cell-specific Egr2 conditional knockout (Egr2^*fl/fl*^CD4Cre^+^: Egr2 CKO) mice and control mice analyzed by FCM. **(b)** CD4^+^ T cells purified from each mouse were analyzed by FCM after 48 h stimulation with the indicated concentration of anti-CD3ε/anti-CD28 antibodies. Histograms are the representative of 3 independent experiments.

To examine whether expression of PD-L1 in LAG3^+^ Tregs was dependent on Egr2, we employed T cell-specific Egr2 conditional knockout mice (Egr2^*fl/fl*^CD4Cre^+^: Egr2 CKO). Unexpectedly, under physiological conditions, LAG3^+^ Tregs from Egr2 CKO mice expressed PD-L1 protein to the same extent as the littermate control mice (Fig. 2a). It has been reported that TCR stimulation induces the upregulation of PD-L1 in CD4^+^ T cells.^8^ Here, we found that the expression levels of PD-L1 in Egr2-deficient CD4^+^ T cells stimulated with anti-CD3ε and anti-CD28 antibodies *in vitro* was not affected (Fig. 2b). These results indicate that Egr2 is not necessary for the induction of PD-L1 in either LAG3^+^ Tregs or activated CD4^+^ T cells, suggesting the involvement of another PD-L1 induction mechanism in CD4^+^ T cells.

### Klf1 induces PD-L1 expression in CD4^+^ T cells independent of Egr2

**Figure 3:**
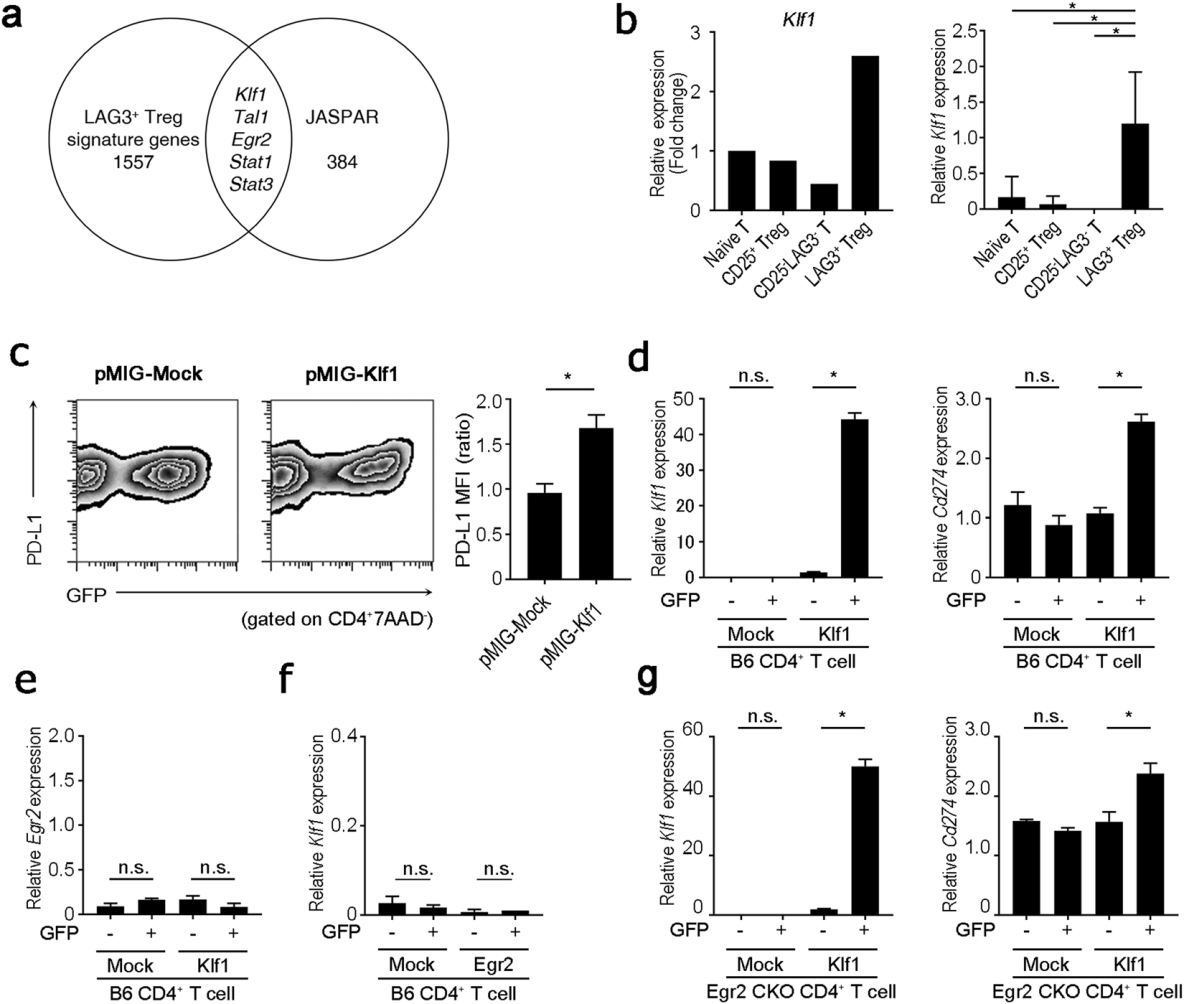
Egr2-independent induction of PD-L1 expression in CD4^+^ T cells by ectopic expression of Klf1. **(a)** Venn diagram representing the genomic overlap between LAG3^+^ Tregs specific transcription factors from microarray analysis of CD4^+^ T cell subsets from ArrayExpress database (E-MEXP-1343) and PD-L1-inducible candidate genes predicted by JASPAR database. **(b)** Gene expression levels of *Klf1* in CD4^+^ T cells. *Klf1* gene expression in indicated T cell subsets relative to unstimulated naïve CD4^+^ cells using micro array data set as in (a) (left) and relative to *Actb* mRNA of each indicated T cell subset confirmed by qRT-PCR (right). *p < 0.001 (Bonferroni’s multiple comparison test) **(c)** Evaluation of PD-L1 expression in *Klf1* gene-transduced CD4^+^ T cells. Klf1 cDNA was inserted into the retrovirus vector, pMIG with a GFP reporter gene. pMIG-Mock or pMIG-Klf1 was transfected into CD4^+^ T cells. MFI ratio represents the PD-L1 MFI signals of GFP negative versus GFP positive. **(d)** GFP^+^ and GFP^-^ cells in (c) were fractionated and mRNA expression of *Cd274* (encoding PD-L1) was evaluated by qRT-PCR (n = 3). **(e**,**f)** *Egr2* and *Klf1* mRNA expression was evaluated by qRT-PCR using the cells transfected in (c) and Fig. 1d. (**g**) *Klf1* and *Cd274* mRNA expression in *Klf1* gene-transduced CD4^+^ T cells from Egr2^*fl/fl*^CD4Cre^+^ mice was evaluated by qRT-PCR (n = 3). *p < 0.05; n.s.: not significant (unpaired two-tailed Student’s *t*-test) Plots are representative of 3 independent experiments.

To analyze other transcription factors that induce PD-L1 expression, we selected 1562 LAG3^+^ Treg-specific genes from microarray data of T cell subsets, including freshly isolated naïve T cells, CD25^+^ Tregs, LAG3^+^ Tregs and CD4^+^CD25^−^CD45RB^low^LAG3^−^ memory T cells registered in the ArrayExpress database (access number E-MEXP-1343)^14^. Furthermore, we performed a transcription factor binding site enrichment analysis using the JASPAR Database^18^, an open-access database for eukaryotic transcription factor binding profiles. Then, we combined a transcriptomics and *in silico* binding prediction approach to identify PD-L1-inducible genes in LAG3^+^ Tregs. As shown in Figs 3a, 5 genes overlapped in these two data sets: signal transducer and activator of transcription 1 (*Stat1*), *Stat3*, *Klf1*, T-cell acute lymphocytic leukemia protein 1 (*Tal1*), and *Egr2*. Stat1 and Stat3 directly bind to the promoter region of the *Cd274* gene^19,20^. Intriguingly, transcription factor genes *Klf1* and *Tal1* were picked up as novel candidate PD-L1-inducible genes. To validate the expression profiles obtained by microarray data, qRT-PCR was performed on *Klf1* and *Tal1* genes, and both genes were characteristically upregulated in LAG3^+^ Tregs (Figs 3b and S1). PD-L1 protein was induced in pMIG-*Klf1* transfected cells, but not pMIG-*Tal1* transduced cells (Figs 3c,d, and S2). We next focused on the mechanism by which Klf1 induced PD-L1. We examined the dependency of *Egr2* expression on Klf1-mediated induction of PD-L1 in CD4^+^ T cells. Expression of *Egr2* was not induced in *Klf1*-transduced CD4^+^ T cells (Fig. 3e). On the other hand, *Klf1* was not induced in *Egr2*-transduced CD4^+^ T cells (Fig. 3f). Moreover, CD4^+^ T cells from Egr2 CKO mice expressed PD-L1 when Klf1 was overexpressed (Fig. 3g). Although we and others have shown that Egr3 compensates for the function of Egr2 during Egr2 deficiency^17,21^, PD-L1 expression was induced by Klf1 in CD4^+^ T cells from Egr3^*fl/fl*^Egr2^*fl/fl*^CD4Cre^+^ (Egr2/3 DKO) mice (Fig. S3). These results clearly indicate that Klf1 induces PD-L1 in CD4^+^ T cells independently of Egr2/Egr3 expression.

### Marked induction of the *Klf1* gene in CD4^+^ T cells modulates the gene expression profile

**Figure 4:**
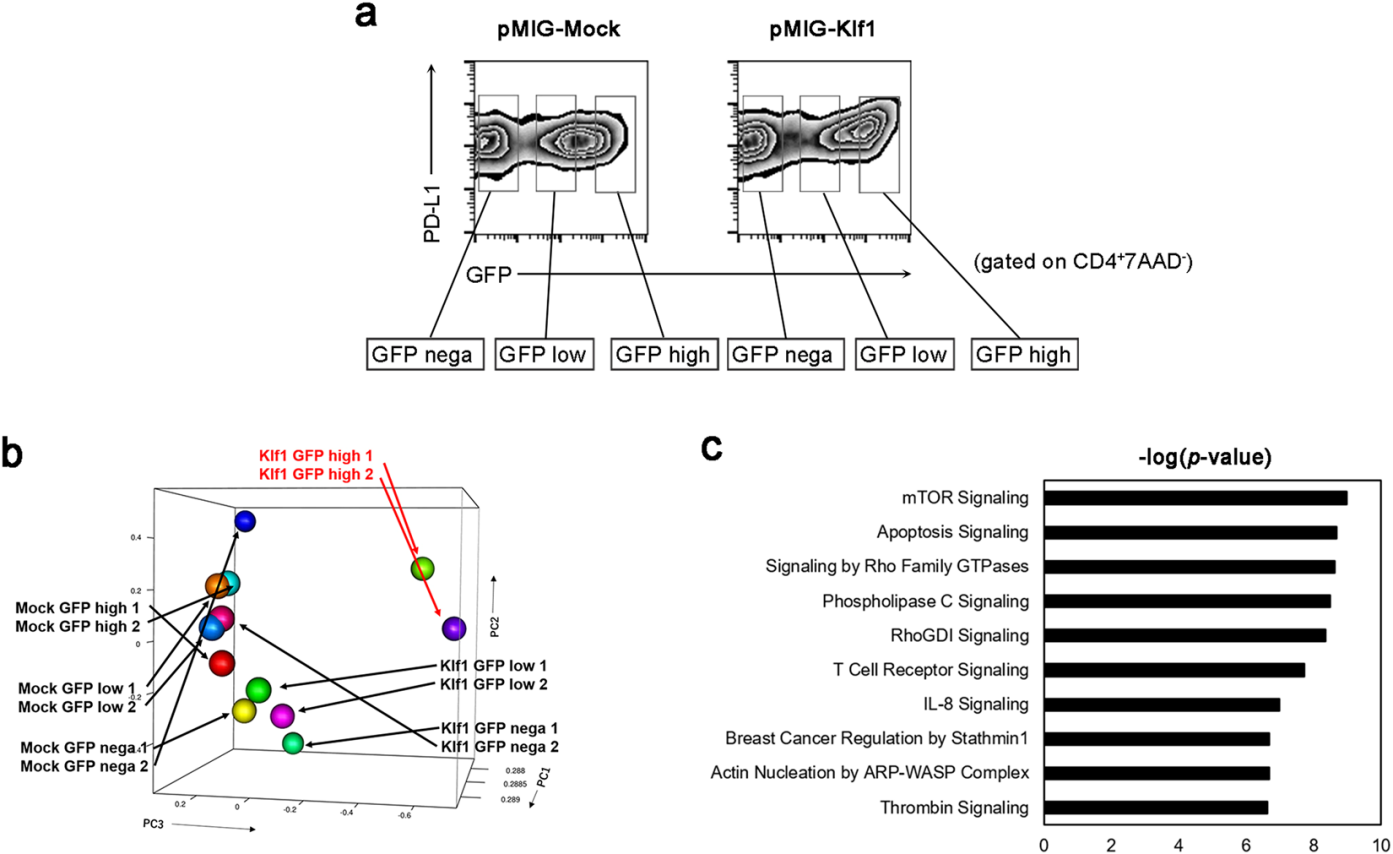
Comprehensive analysis of gene expression profiles in *Klf1*-transduced CD4^+^ T cells. **(a)** Sorting strategy for next generation sequencing (NGS) of *Klf1*-gene-transduced CD4^+^ T cells. Splenocytes from WT mice were transfected with pMIG-Mock or pMIG-*Klf1* vector according to their GFP expression. **(b)** Principal component analysis plots for NGS data of the indicated CD4^+^ T cell subsets as in (**a**,**c)** Differentially expressed genes of NGS data sets in (**b**) were analyzed by pathway analysis tool Ingenuity Pathway Analysis (IPA) software. The top 10 most significantly enriched canonical pathways in IPA are shown.

To clarify the mechanism by which PD-L1 is induced by Klf1 in CD4^+^ T cells, changes in the gene expression profile in pMIG-*Klf1*- or pMIG-Mock-transduced cells were divided into three groups according to GFP expression levels and analyzed by RNA sequencing (Fig. 4a). Principal component analysis (PCA) of these cells showed that *Klf1*-transduced cells (Klf1 GFP^high^) had unique characteristics compared with the other cells (Fig. 4b). Pathway analysis of the differentially expressed genes (DEGs) was conducted using Ingenuity Pathways Analysis software (IPA). IPA analysis showed candidate intracellular signal transduction pathways induced by Klf1, which include mammalian target of rapamycin (mTOR) signaling pathway as the top canonical pathway, indicating that mTOR signaling played a crucial role in Klf1-mediated PD-L1 expression (Fig. 4c).

### Inhibition of the PI3K-mTOR signaling pathway decreases Klf-1-mediated induction of PD-L1 expression in CD4^+^ T cells

**Figure 5:**
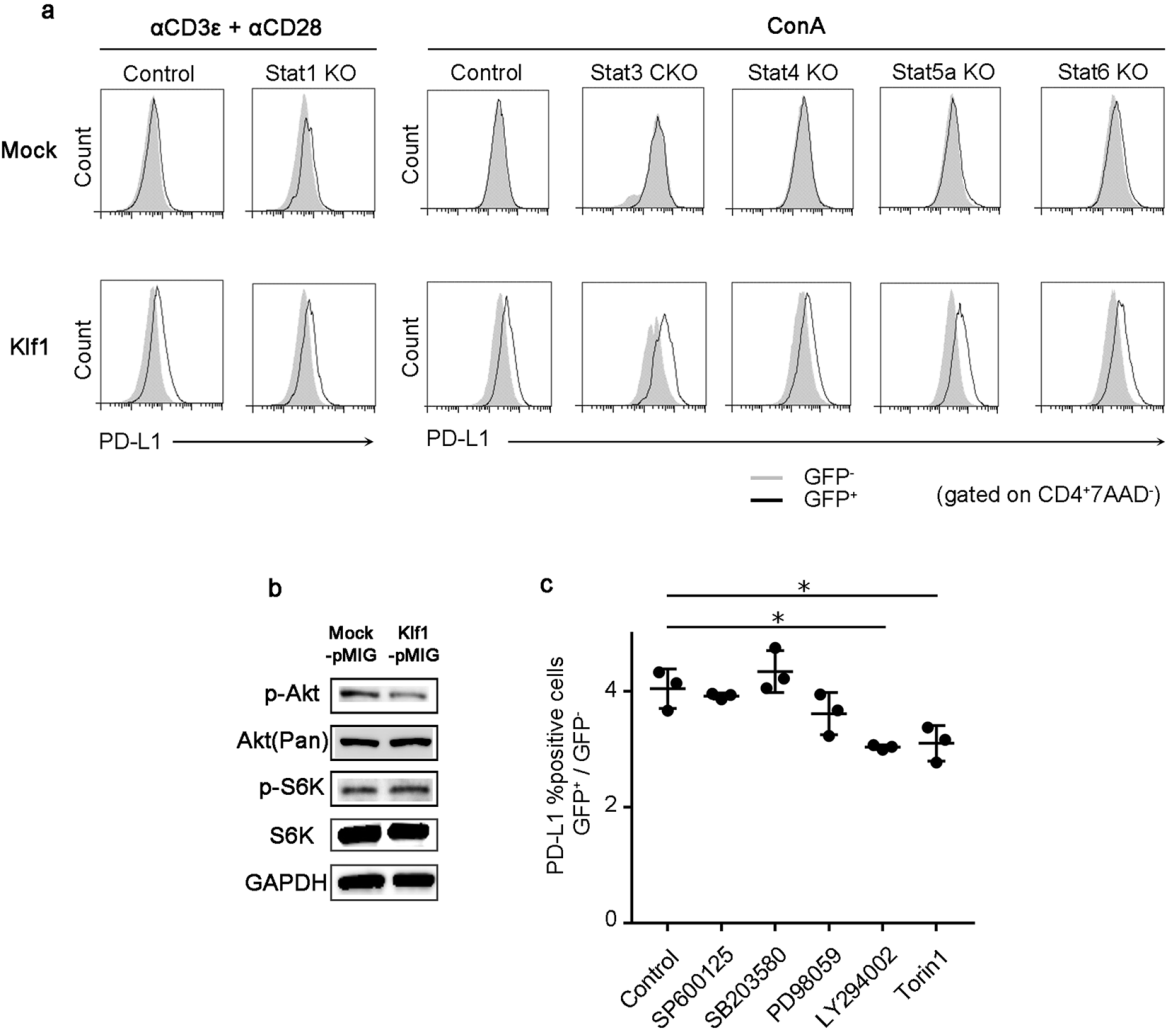
Klfl-mediated PD-L1 induction requires the activation of PI3K-mTOR signaling pathway. **(a)** Splenocytes from each Stat-deficient or WT mouse were transfected with a pMIG-Mock or pMIG-*Klf1* vector. PD-L1 expression levels were assessed by FCM 48 h after the transfection. In the Stat1 KO mouse, anti-CD3ε/anti-CD28 stimulation was used whereas Concanavalin A (ConA) stimulation was used for the other KO mice. **(b)** Phosphorylation of Akt and S6K was analyzed by Western blotting. The indicated blots were derived from the same experiments. Full length blots are presented in Supplementary Figure S4. **(c)** Splenocytes from B6 mice were transfected with pMIG-Mock or pMIG-*Klf1* and the indicated inhibitors were added 24 h later. Then, cells were cultured for 48 h and PD-L1 expression was evaluated by FCM. The proportion of cells expressing PD-L1 at high levels was compared between the GFP-positive and GFP-negative groups. (n = 3). Control: without inhibitor; SP 600125: JNK1/2/3 inhibitor (5 μM); SB 203580: MAPK-p38 inhibitor (20 μM); PD 98059: MEK/ERK inhibitor (50 μM); LY 294002: PI3K inhibitor 20 μM); Torin: mTOR inhibitor (200 nM). *p < 0.01 (Bonferroni’s multiple comparison test). Plots are representative of 3 independent experiments.

Consistent with our own analysis (Fig. 3a), previous reports showed that Stat1 and Stat3 directly bind to the promoter region of *Cd274*^19,20,22,23^, suggesting the important role of STATs signaling in the induction of PD-L1. The induction mechanism of PD-L1 is cell-type dependent (see Discussion). To clarify the nature of the Klf1-mediated PD-L1 induction mechanism, we employed Stat1 KO mice, T cell-specific Stat3 KO mice (Stat3^*fl/fl*^CD4Cre^+^: Stat3 CKO), Stat4 KO mice, Stat5a KO mice, Stat6 KO mice and wild-type (WT) mice. Unexpectedly, the expression levels of PD-L1 on *Klf1*-transduced CD4^+^ T cells were not affected by Stat1, Stat3, Stat4, Stat5a nor Stat6 deficiency, indicating that these STATs do not regulate Klf1-mediated upregulation of PD-L1 in CD4^+^ T cells (Fig. 5a).

The PI3K-Akt-mTOR signaling pathway is activated in TCR-stimulated T cells and affects the fate of Th cells and Tregs^24–27^. Our IPA analysis of DEGs in Klf1-transduced cells suggested the PI3K-Akt-mTOR signaling pathway is involved in the Klf1-mediated induction of PD-L1. To verify this hypothesis, PI3K-Akt-mTOR cellular signaling pathways were evaluated by Western blot analysis. Unexpectedly, there was no difference in the phosphorylation levels of Akt and its downstream effector (S6K protein) between *Klf1*-transduced CD4^+^ T cells and mock-transduced CD4^+^ T cells (Figs 5b, S4). To examine whether PD-L1 expression was dependent on active PI3K-AKT-mTORsignaling, *Klf1*-transduced CD4^+^ T cells were treated with pharmacologic inhibitors, including a JNK1/2/3 inhibitor (SP600125), a p38 inhibitor (SB203580), an MEK inhibitor (PD9805981), a PI3K inhibitor (LY294002) and an mTOR inhibitor (Torin1). Treatment with LY294002 and Torin1 significantly reduced the Klf1-mediated induction of PD-L1 (Fig. 5c). These results indicate that a basal level PI3K-Akt-mTOR signaling is necessary for PD-L1 induction by Klf1 in CD4^+^ T cells, suggesting that Klf1 and the PI3K-Akt-mTOR signaling pathway might cooperate to induce PD-L1 expression on CD4^+^ T cells.

## Discussion

Previous work has established that PD-L1 has critical functions as an immunosuppressive cell surface molecule. However, the precise mechanisms by which PD-L1 is induced on CD4^+^ T cell have long remained elusive. Here, we showed that the expression of PD-L1 on CD4^+^ T cells is induced by Klf1 and Egr2. Klf1 was predicted by *in silico* analysis from the gene expression profile of Egr2-expressing LAG3^+^ Tregs that characteristically express PD-L1. Klf1-mediated PD-L1 induction by CD4^+^ T cells, and it was partially dependent on the PI3K-AKT-mTOR signaling pathway, but independent of Egr2 and STATs signaling. Our findings have important implications for understanding the molecular mechanisms underlying cell-mediated immune tolerance modulated by CD4^+^ T cells that express PD-L1.

Interaction of PD-1 and its ligand PD-L1 or PD-L2 negatively regulates intracellular signaling by recruiting protein tyrosine phosphatases, SHP-1 and SHP-2^28^. PD-L1 KO mice show enhanced Th1 responses, exacerbation of experimental autoimmune encephalomyelitis and autoimmune hepatitis^29,30^. In human, the clinical relevance of genetic polymorphisms in the human *CD274* gene, encoding PD-L1, has been reported in several autoimmune diseases, such as Addison’s disease and Graves’ disease^31,32^. On the other hand, deficiency of PD-L2 results in orally administered antigen intolerance^33^, indicating different roles for PD-L1 and PD-L2 in immunity and pathogenesis. Although the expression of PD-L2 is restricted mainly to activated dendritic cells and macrophages^6^, PD-L1 is expressed in various immune cells, including activated CD4^+^ T cells and Treg subsets. PD-1/PD-L1 interaction is also known as an inhibitory mechanism in CD25^+^ Tregs^13^ and LAG3^+^ Tregs. These findings clearly suggest that PD-L1 expression on CD4^+^ T cells is important for maintenance of immune tolerance.

We previously reported that forced expression of Egr2 in CD4^+^ T cells provides the functional traits of LAG3^+^ Treg, such as induced expression of Prdm1, IL-10 and LAG3^14^. However, the role of Egr2 in induction of PD-L1 in CD4^+^ T cells has not been clarified. We demonstrated that Egr2 is not required for PD-L1 expression on CD4^+^ T cells. It has been reported that among the 4 Egr family members, Egr3 compensates for the function of Egr2 when the latter is deficient^21^. We reported that expression of latent TGF-β binding protein (Ltbp)-3 was maintained by both Egr2 and Egr3 and was required for TGF-β3 production from LAG3^+^ Tregs^17^. However, T cells that were doubly-deficient for Egr2 and Egr3 were not affected in their Klf-1-mediated induction of PD-L1, indicating that Klf-1 induces PD-L1 in CD4^+^ T cells via an Egr2/Egr3- independent manner.

These findings indicate a possibility of the involvement of another Egr2-independent PD-L1 induction mechanism in CD4^+^ T cells, and we focused on *Klf1* based on the expression on LAG3^+^ Tregs. The Klf family is a transcription factor group composed of 17 genes, many of which are important for immune homeostasis. Klf1 is also called erythroid KLF (EKLF) and is predominantly expressed in erythroid progenitor cells. Klf1 has important functions in a variety of mechanisms, including gene activation and repression, regulation of chromatin arrangement, transcription initiation and elongation^34^. Klf1 binds to the CACCC site in the promoter region of the β-globin gene^35,36^. In humans, KLF1 also plays a role in transcribing the β-globin gene. A mutation in the CACCC region causes β-thalassemia^37,38^. Although Klfl is characterized primarily as a transcriptional activator, it can also act as a repressor that interacts with the components of the corepressor Sin3A and the NuRD complex^39,40^. KLF1 participates in the repression of the fetal γ-globin genes in human adult erythroid progenitors by regulating the expression of BCL11A. However, the role of Klf1 in the immune system has not been clarified since deficiency of Klf1 results in embryonic lethality due to congenital thalassemia^41^. In the present study, we revealed a new immunological function of Klf1 in PD-L1 induction in CD4^+^ T cells that was achieved in an Egr2/Egr3-independent manner.

The induction mechanism of PD-L1 appears to depend on the cell type. Extracellular stimuli and intracellular signaling mechanisms that contribute to the expression of PD-L1 have been studied mainly in tumor cells. A cell line derived from lung cancer cells (A549) expresses PD-L1 following interferon (IFN)-γ stimulation, but PD-L1 expression is attenuated by a Jak/Stat inhibitor^42^. Mouse and human T cell lymphoma cells express PD-L1 in the steady state, and Stat3 binds to the promoter region of the *Cd274* gene encoding PD-L1^19,22,23^. In Hodgkin’s lymphoma cells, expression of PD-L1 was reduced through inhibition of STAT3 expression by a MAPK inhibitor^20^. PD-L1 expression is promoted by Toll-like receptor 4- (TLR4) stimulation in human multiple myeloma-derived plasma cells, and this PD-L1 expression is dependent on the mitogen- activated protein kinase (MAPK) signal pathway^43^. In human dermal fibroblasts, phosphorylation of ERK1/2 and PI3K induces expression of PD-L1^44^. On the other hand, in dendritic cells, Stat4- or Stat6-deficiency does not affect PD-L1 expression^45^. Although accumulating evidence has revealed the induction mechanisms of PD-L1 in various cell types, that of PD-L1 in CD4^+^ T cells has yet to be determined. In the present study, we revealed the unique signaling pathway that cooperates to induce PD-L1 expression in CD4^+^ T cells. Although our *in silico* analysis suggests the involvement of Stat1 and Stat3, deficiencies of various STATs, including Stat1 and Stat3, did not affect Klf1-induced PD-L1 expression levels. Thus, it appears that Stat1 and Stat3 are not necessary for the Klf1-mediated PD-L1 induction in CD4^+^ T cells.

Previous studies revealed that the PI3K-Akt-mTOR pathway plays a critical role in PD-L1 expression. In murine squamous cell carcinoma cells, breast cancer, prostate cancer^46^ and human glioma cells, inactivation of phosphatase and tensin homolog deleted from chromosome 10 (PTEN) promotes PD-L1 expression through the PI3K-Akt pathway and mTOR activation^47,48^. In human non-small cell lung cancer, PD-L1 is also induced by mTOR activation^49^. In T cells, Akt-mTOR signaling is a key driver of activation, differentiation and function. T cells possess a unique metabolic profile and a corresponding set of mTOR signal requirements. In the present study, we revealed that Klf1-mediated PD-L1 induction in CD4^+^ T cells is dependent on the PI3K-Akt-mTOR signaling pathway.

In recent years, treatments focused on PD-1/PD-L1 signaling have been clinically applied to the field of tumor immunity, but this approach has not been expanded to autoimmune diseases. Targeting PD-L1 and PD-1 is a potentially novel treatment strategy, and it has attracted attention. In the field of tumor immunity, several monoclonal antibodies against PD-1 or PD-L1 have been developed and clinical studies initiated. These include treatments with the anti-PD-1 antibody Nivolumab and the anti-PD-L1 antibody Avelumab for treatment of skin cancer and non-small cell lung cancer^50–52^. Tumor immunity and autoimmunity are inseparable, and autoimmune side effects are observed in about 5% of subjects treated with anti-PD-1 antibody and anti-PD-L1 antibody^53,54^. Pancreatic islet cells highly express PD-L1 in nonobese diabetic (NOD) mice, and exacerbation of the disease is observed by administration of anti-PD-1 antibody or anti-PD-L1 antibody, whereas no change was observed with anti-PD-L2 antibody^55^. Similar trends are also seen in a contact hypersensitivity model^56^ and a coronary artery lesion model following heart transplantation^57^. These reports indicate that PD-1/PD-L1 interaction is a novel therapeutic target for autoimmune diseases.

Here, it was shown that Klf1 and Egr2, transcription factors specifically expressed in LAG3^+^ Tregs, induce PD-L1 expression in CD4^+^ T cells independent of each other. These novel findings shed light on the understanding of PD-L1-mediated immune tolerance and suggest the potential therapeutic targeting of Klf1 and Egr2 in the treatment of autoimmune diseases and malignancies.

## Materials and Methods

### Mice

C57BL/6J (B6) mice were purchased from Japan SLC. 129S6/SvEv-Stat1^*tm1Rds*^ (Stat1 KO) mice and CD4Cre^+^ mice were purchased from Taconic Biosciences (Germantown, NY, USA). Stat3^*fl/fl*^CD4Cre^+^ (Stat3 CKO) mice, C129S2-Stat4^*tm1Gru*^/J (Stat4 KO) mice, C.129 S (B6) -Stat5a^*atm1Mam*^/J (Stat5a KO) mice and C.129S2-Stat6^*tm1Gru*^/J (Stat6 KO) mice were purchased from Oriental Yeast Co. (Tokyo, Japan). T cell-specific Egr2 conditional knockout (Egr2^*fl/fl*^CD4Cre^+^: Egr2 CKO) mice were produced by crossing Egr2^*fl/fl*^ mice (provided from Patrick Charnay (INSERM, France)) and CD4Cre^+^ mice. Egr2/3 DKO mice (Egr2^*fl/fl*^Egr3^*fl/fl*^CD4Cre^+^) were generated by crossing Egr2 CKO mice with Egr3^*fl/fl*^ mice^17^. Control mice were littermates of Egr2^*fl/fl*^, CD4Cre^+^ or wild-type (wild type: WT) mice. All mice were housed in a specific pathogen-free environment. For the experiments, mice were at least 6–8 weeks of age. Mice of 24–28 weeks of age were used for analysis of LAG3^+^ Tregs. All animal experiments were approved by the ethics committee of the University of Tokyo Institutional Animal Care and Use Committee, and all experiments were conducted based on the approved experimental plan according to the guidelines of the University of Tokyo.

### Reagents

Anti-CD3ε antibody (145-2C11), anti-CD28 antibody (37.51), Fc blocking antibody (anti-CD16/CD32 antibody), FITC- anti-CD45RB antibody (16A) and PE- anti-LAG3 antibody (C9B7W) were obtained from BD Biosciences (San Jose, CA, USA). Biotinylated anti-CD8a antibody (53-6.7), biotinylated anti-CD11c antibody (HL3), PE-anti-CD25 antibody (PC61), APC- anti-CD25 antibody (3C7), biotinylated anti-PD-L1 antibody (10 F.9G2), avidin (SA)-APC antibody, APC/Cy7-anti-CD4 antibody (RM4-5) and 7-AAD were purchased from BioLegend (San Diego, CA, USA). Biotinylated anti-CD19 antibody (1D3) was purchased from eBioscience (Frankfurt am Main, Germany). Streptavidin microbeads were purchased from Miltenyi Biotec (Bergisch Gladbach, Germany and recombinant murine IL-2 was purchased from R&D Systems (Minneapolis, MN, USA). For cultivation, T cells were incubated in RPMI 1640 supplemented with 10% fetal bovine serum (FBS), 100 μg/mL L-glutamine, 100 U/mL penicillin, 100 μg/mL streptomycin and 50 μM 2-mercaptoethanol (all purchased from Sigma). Packaging Cells Plat-E were prepared by culturing in Dulbecco’s modified Eagle’s medium (DMEM) containing 10% FBS, 100 μg/mL L-glutamine, 100 U/mL penicillin, 100 μg/mL streptomycin, 1 μg/mL puromycin and 10 μg/mL blasticidin.

### Flowcytometry

Mouse spleens were treated with type IV collagenase (Sigma, St. Louis, USA), and CD4^+^ T cells were collected using a magnet-assisted cell sorting (MACS) system (Miltenyi). Cells were magnetically labeled using biotinylated anti-CD8a, CD11c, and CD19 antibodies and streptavidin microbeads, and the negative fraction that passed through the MACS LS column (Miltenyi) was collected as CD4^+^ T cells. LAG3^+^ Tregs were collected by negative selection using biotinylated anti-CD45 RB antibody. Collected cells were labeled using FITC-anti-CD45RB antibody, PE-anti-LAG3 antibody, APC-anti-CD25 antibody, APC/Cy7-anti-CD4 antibody after blocking Fc receptors. Cell collection was performed with CellQuest (BD Biosciences) using a FACS Vantage flow cytometer (BD Biosciences), and analysis was performed by FlowJo (Tree Star, USA). The purity of cells concentrated by MACS was 90% or more, and the purity of cells collected by flow cytometry (FCM) was 99%.

### Quantitative real-time PCR

RNA was extracted using an RNeasy Micro kit (QIAGEN, Germantown, USA) and reverse transcribed into cDNA using SuperScript III and Random Primers (both Invitrogen). Quantitative RT-PCR (qRT-PCR) was performed with CFX Connect (Bio-Rad) using QuantiTect SYBR Green PCR Kit (QIAGEN). The RNA expression level of the target gene was evaluated relative to β-actin (*Actb*). Sequences of primers are shown in Supplementary Table S1.

### Microarray analysis

Microarray analysis was performed as described elsewhere^14^. Briefly, the gene expression profile of murine CD4^+^ T cell subsets was obtained from an ArrayExpress database, access number E-MEXP-1343 data. Background adjustment and standardization were performed using Bioconductor’s Mas5 package in statistical software R. Hierarchical clustering was performed in GeneSpring GX (Agilent Technologies, Santa Clara, CA, USA) for genes whose expression was enhanced 1.5-fold compared to CD4^+^CD25^−^CD45RB^high^ T cells (naïve CD4^+^ T cells), or expression decreased at least 0.6-fold. Gene expression level was evaluated as the expression level relative to naïve CD4^+^ T cells.

### T cell isolation

T cells were isolated from mouse splenocytes and then collected in the MACS system using a naïve CD4^+^ T cell isolation kit (Miltenyi Biotec) as follows. Mouse splenocytes were harvested, passed through a 70 μm cell strainer (BD Bioscience), and hemolyzed using an ACK lysis buffer. A solution containing antibodies against a biotin-antibody cocktail (anti-CD8a, anti-CD11b, anti-CD11c, anti-CD19, anti-CD25, anti-CD45R (B220), anti-CD49b (DX5), antiCD105, anti-MHC Class II, anti-Ter119 and anti-CD16/CD32 (Fcg III/II Receptor) was added and reacted at 4 °C for 10 min. Then, 200 μL of anti-biotin microbeads were added and reacted at 4 °C for 15 min, and T cells were separated by negative selection using an LS column. After separation, staining was carried out using anti-CD44 and anti-CD62L antibodies and analyzed by FCM; the purity was >90%.

### Gene transduction using retroviral vectors

DNA encoding Egr2 protein (NM-010118) was isolated from mouse T cell cDNA and subcloned into the retroviral vector pMIG. The full-length mRNA of mouse *Klf1* (NM-010635) was synthesized by Takara Bio and subcloned into the retroviral vector pMIG. PMIG-Mock, pMIG-*Egr2* and pMIG-*Klf1* were transfected into packaging cells (Plat-E) using the Extreme Gene 9 transfection reagent (Roche) and the virus-containing culture supernatant was collected. Mouse splenocytes were cultured for 48 h in the presence of Concanavalin A (ConA) (10 μg/mL) (Sigma) and IL-2 (50 μg/mL) to prepare ConA blasts, and gene transduction was conducted using a retroviral supernatant. Specifically, a viral supernatant was added to a Falcon 24-well plate coated with a recombinant human fibronectin fragment CH296 (RetroNectin, Takara), centrifuged at 1200 × g at room temperature for 3 h, and the same operation was repeated three times. After removal of the viral supernatant, ConA blasts were transferred to a plate and cultured in an incubator for 48 h for viral infection. Cultured cells were collected and used for subsequent experiments. In intracellular signal suppression experiments, JNK1/2/3 inhibitor SP600125 (5 μM, Wako Pure Chemical Industries, Ltd., Osaka, Japan), PI3K inhibitor LY294002 (20 μM, Wako), MAPK (p38) inhibitor SB203580 (20μM, Cayman), the MAPK (MEK) inhibitor PD98059 (40 μM, Cayman) or mTOR inhibitor Torin1 (200nM, Cayman) were added 24 h after initiation of infection, and cultured for further 48 h before FCM analysis.

### Cell culture

Naïve CD4^+^ T cells were isolated from splenocytes from Egr2 CKO mice and Egr2^*fl/fl*^, Egr2^*fl/+*^, CD4Cre^+^ (control) mice using the mouse naïve CD4^+^ T cell isolation kit (Miltenyi Biotech) with a MACS system. The cells were cultured for 72 h on anti-CD3ε antibody (2–10 μg/mL) and anti-CD28 (5 μg/mL) antibody-coated 96-well flat bottom plates, followed by FCM analysis.

### RNA-sequencing

CD4^+^ T cells that were transfected with retroviral vectors were divided into 3 fractions according to GFP fluorescence (strongly positive, weakly positive and negative) using a FACSVantage flow cytometer. Total RNA was then extracted. Sequence libraries were prepared according to standard methods using Smart-seq2 (Illumina), and they were analyzed with a next generation sequencer, a MiSeq system (Illumina). Resultant data were mapped to UCSC mm10 using the STAR program, and the read count was calculated using HTSeq. Principal component analysis (PCA) was performed using Prcomp (R package), and visualization in three dimensions was performed by Plot 3D (R package). Differentially expressed genes were analyzed using Ingenuity Pathway Analysis (QIAGEN).

### Western blotting

CD4^+^ T cells transfected with retroviral vectors were cultured in lysis buffer (50 mM Tris HCL, 0.15 M NaCl, 1% Triton-X, 1 mM EDTA) supplemented with Halt protease inhibitor cocktail kit (1:100; Thermo Fisher), an equal amount of 2 × Laemmli sample buffer (Sigma) was added, and the mixture was reacted at 95 °C for 5 min. Total protein was measured using a BCA Protein Assay kit (Pierce). Each sample was electrophoresed on a 7.5% Mini-PROTEAN TGX precast gel (Bio-Rad) and transferred to an Immobilon-PVDF membrane (Millipore). After blocking with 5% bovine serum albumin, the membrane was reacted with anti-phospho-Akt (Thr308) antibody (1:1000), anti-Akt (Pan) antibody (1:1000), anti phospho-S6K1 (Thr389) antibody (1:1000), anti-S6K1 antibody (1:1000) or anti-GAPDH antibody (1:1000) at 4 °C overnight, and then goat anti rabbit IgG-horseradish peroxidase (HRP) (Invitrogen 1:3000) was added at room temperature for 1 h. Signals were detected using ECL Prime or ECL Select (GE Healthcare).

### Statistical analysis

Statistically significant differences were analyzed using GraphPad Prism 7 (GraphPad Software, Inc.). Comparative study of qRT-PCR and mean fluorescence intensity was conducted by Student’s *t*-test in the case of two groups. Comparative studies between multiple groups were conducted by one-way ANOVA and then a multiple comparison test was performed using the Bonferroni method.

### Data availability

Please contact the corresponding author for data requests.

### Accession number

The microarray and RNA-sequencing data are available online at in the ArrayExpress database (access number E-MEXP-1343, E-MTAB-6778).

## Electronic supplementary material

Supplementary information
