# Supplementary material for: Egr2-independent, Klf1-mediated induction of PD-L1 in CD4+ T cells
Source: Sci Rep. 2018 May 4;8:7021. doi: 10.1038/s41598-018-25302-1 (PMC5935736; doi:10.1038/s41598-018-25302-1)
Supplement: Supplementary file 1 — Supplementary information [file 41598_2018_25302_MOESM1_ESM.docx]

**Egr2-independent, Klf1-mediated induction of PD-L1**

**in CD4^+^ T cells**

**Shuzo Teruya^1^, Tomohisa Okamura^1,2,4*^, Toshihiko Komai^1^, Mariko Inoue^1^, Yukiko Iwasaki^1^, Shuji Sumitomo^1^, Hirofumi Shoda^1^,**

**Kazuhiko Yamamoto^1,2,3^, and Keishi Fujio^1,*^**

*1. Department of Allergy and Rheumatology, Graduate School of Medicine, The University of Tokyo, 7-3-1 Hongo, Bunkyo-ku, Tokyo 113-8655, Japan.*

*2. Max Planck–University of Tokyo Center for Integrative Inflammology, The University of Tokyo, 4-6-1 Komaba, Meguro-ku, Tokyo 153-8505 Japan.*

*Max Planck–University of Tokyo Center for Integrative Inflammology, The University of Tokyo, 4-6-1 Komaba, Meguro-ku, Tokyo 153-8505 Japan.*

*3. Laboratory for Autoimmune Diseases, Center for Integrative Medical Sciences, RIKEN, 1-7-22 Suehiro-cho, Tsurumi-ku, Yokohama, Kanagawa, 230-0045, Japan.*

*4.* *Department of Functional Genomics and Immunological Diseases, Graduate School of Medicine, The University of Tokyo, 7-3-1 Hongo, Bunkyo-ku, Tokyo 113-8655, Japan.*

**To whom correspondence should be addressed. E-mail: tomohisa-tky@umin.ac.jp* and *kfujio-tky@umin.ac.jp*

**Supplementary materials**

**Supplementary Figures**


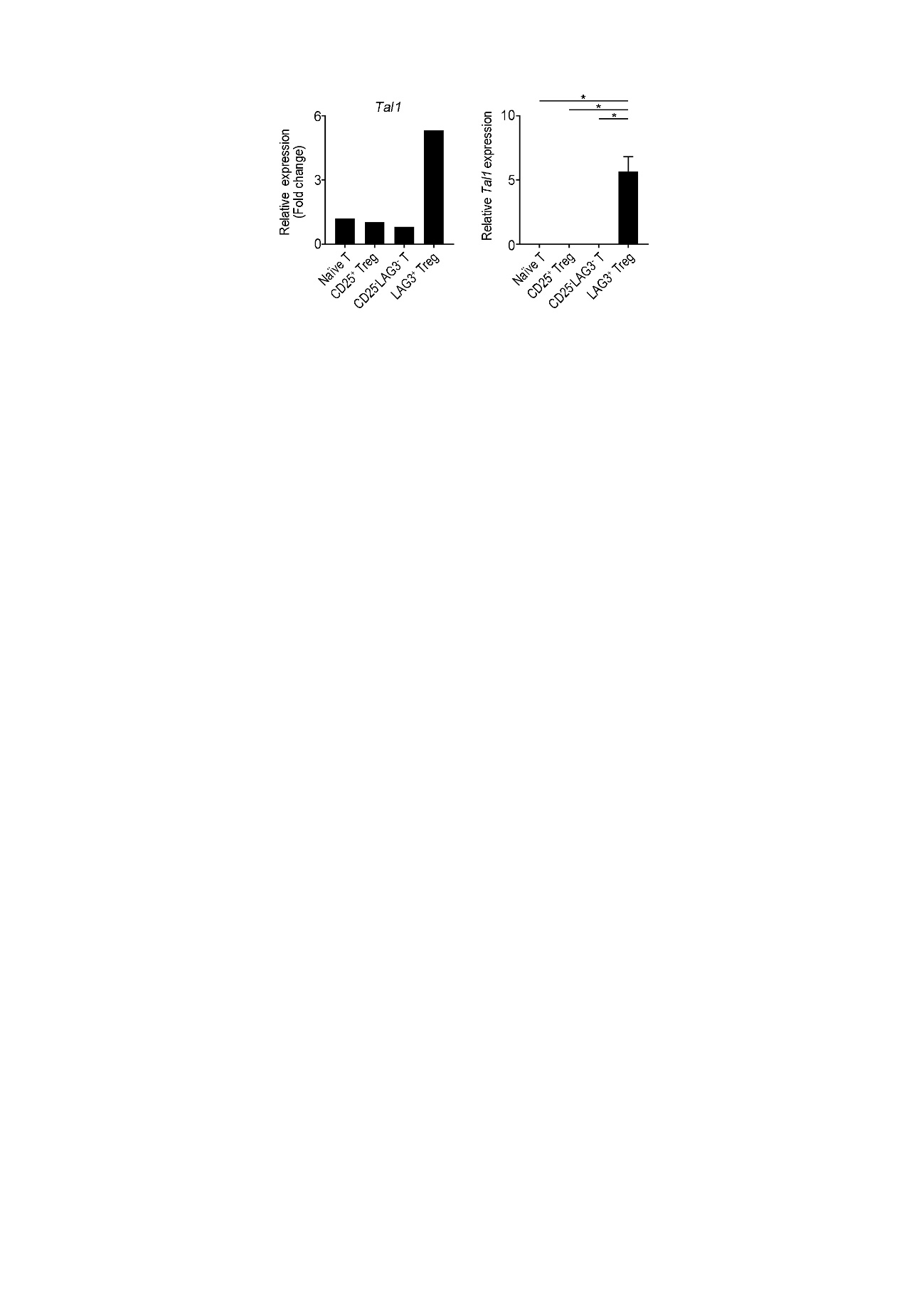


**Figure S1.** Gene expression levels of *Tal1* in CD4^+^ T cells. *Tal1* gene expression of indicated T cell subsets relative to unstimulated naïve CD4^+^ cells using microarray data set as in Fig. 3a (left) and relative to *Actb* mRNA of each indicated T cell subset confirmed by qRT-PCR (right). *, *p* <0.001 (Bonferroni's multiple comparison test)


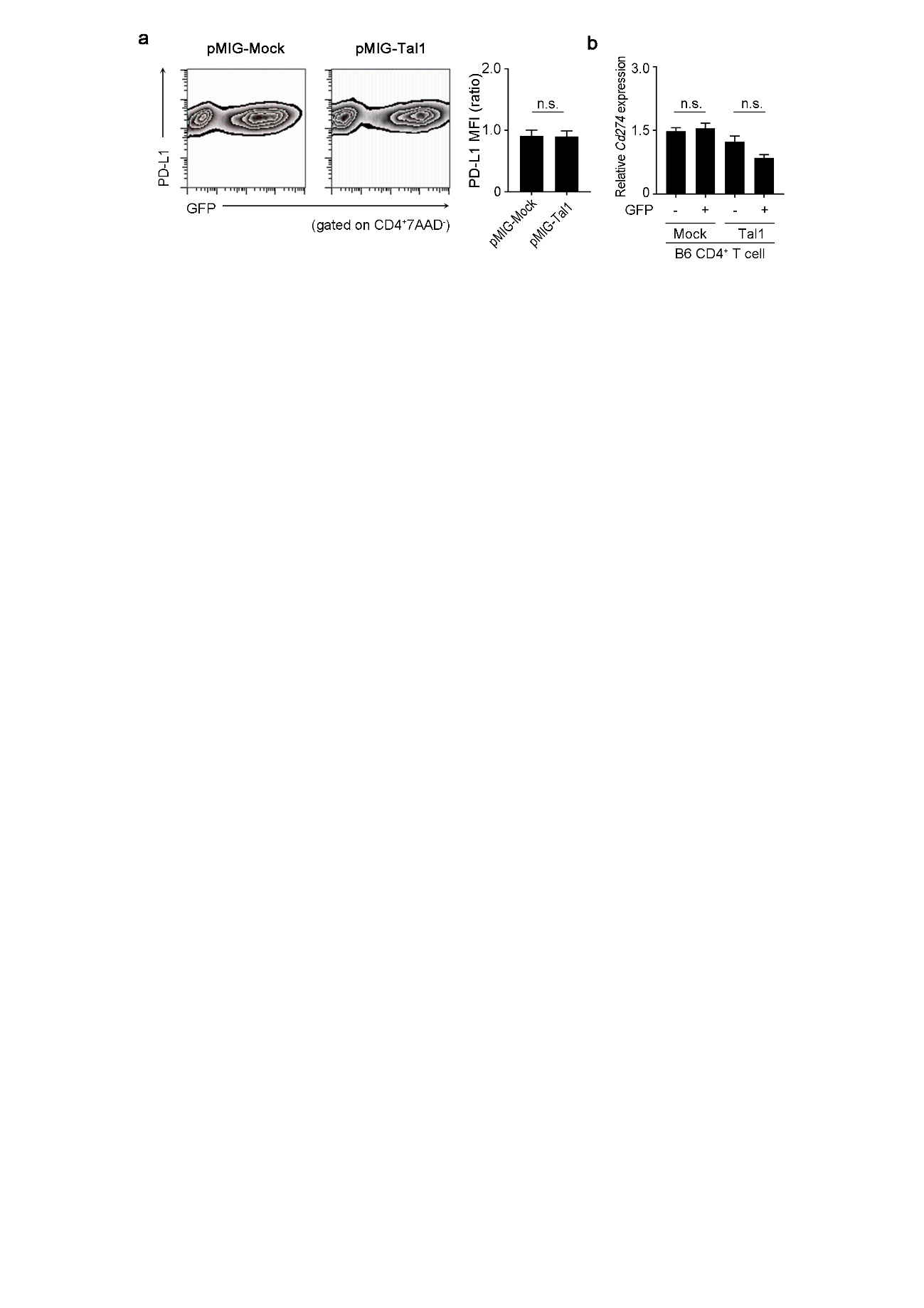


**Figure S2.** Ectopic expression of the *Tal1* gene in CD4^+^ T cells. **(a)** FCM analysis of pMIG-Mock or pMIG-*Tal1*-transduced CD4^+^ T cells. MFI ratio represents the PD-L1 MFI signals of GFP negative versus GFP positive. **(b)** Quantitative PCR analyses of relative *Cd274* gene (encoding PD-L1) expression levels in sorted pMIG-Mock or pMIG-*Tal1*-transduced CD4^+^ T cells populations according to their GFP positivity (n = 3 per group). n.s.: not significant (unpaired two-tailed Student's *t*-test)


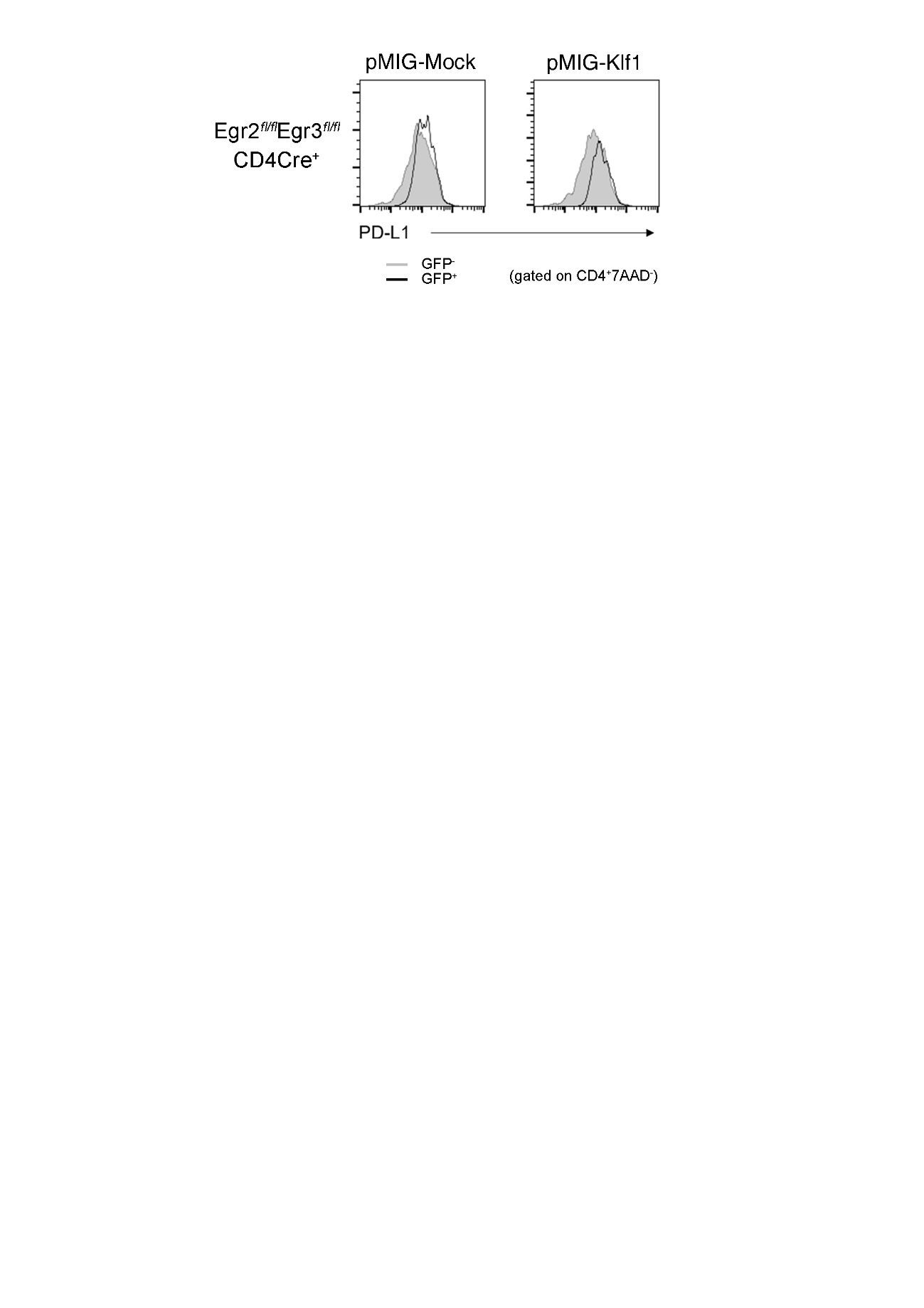


**Figure S3.** PD-L1 expression after ectopic expression of the *Klf1* gene in CD4^+^ T cells from Egr2*^fl/f^*Egr3*^fl/fll^*CD4Cre^+^ mice (Egr2/3 DKO) mice. FCM analysis of pMIG-Mock or pMIG-*Klf1*-transduced CD4^+^ T cells were conducted. Histograms are representative of 3 independent experiments.

**
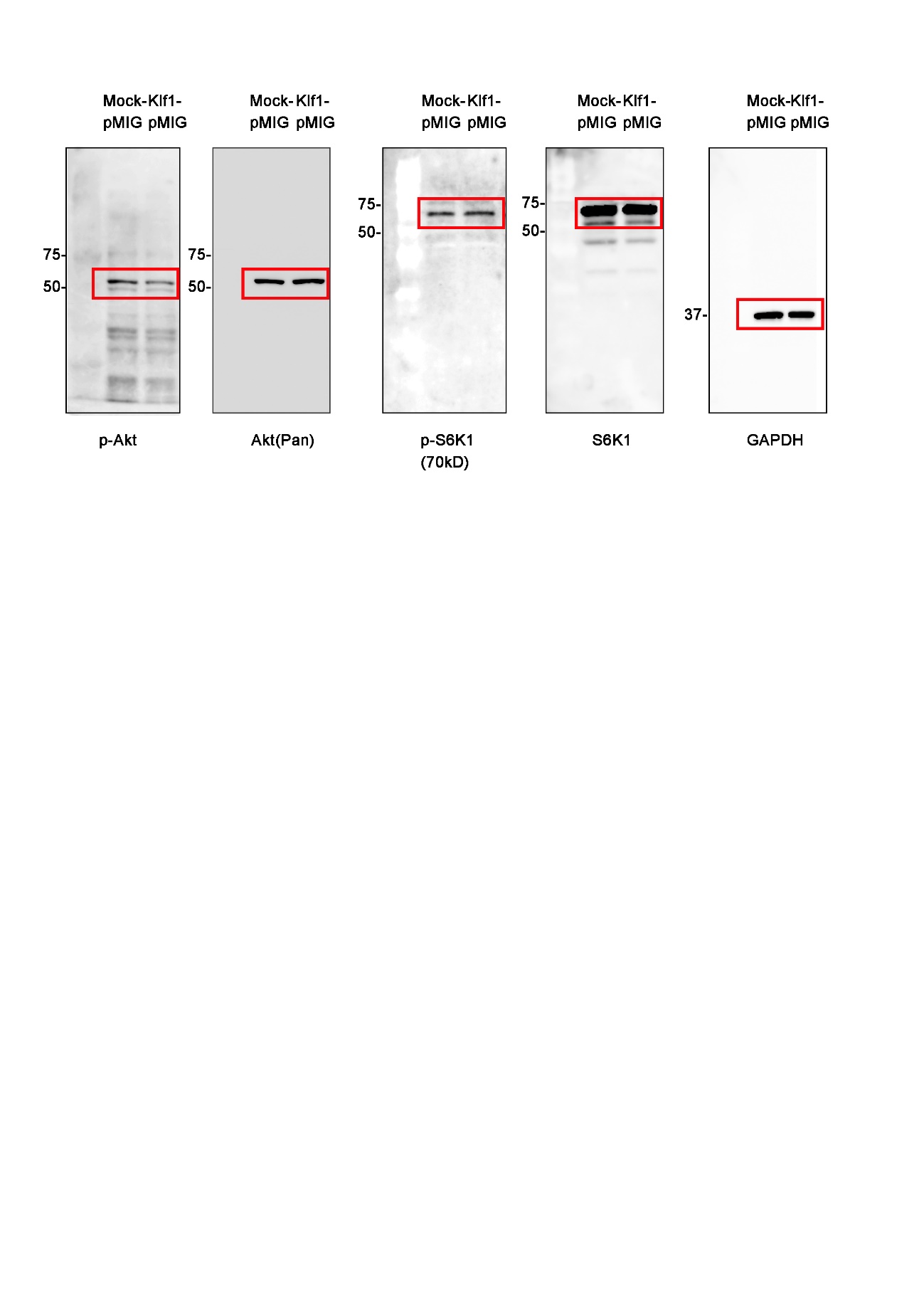
**

**Figure S4.** Full-length blots for **Figure 5b.**

**Supplementary Table**

Table S1. Sequences of primers used for the quantitative real-time PCR.

| Genes | Forward primers | Reverse primers |
| --- | --- | --- |
| *Cd274* | GGAATTGTCTCAGAATGGTC | GTAGTTGCTTCTAGGAAGGAG |
| *Egr2* | AGCCGTTTCCCTGTCCTCTG | GTCCCTCACCACCTCCACTT |
| *Klf1* | ACATACGTGCGGGCACGAAGG | CGTCTGAGCGAGCGAACCTCC |
| *Actb* | AGAGGGAAATCGTGCGTGAC | CAATAGTGATGACCTGGCCGT |
